# Disparities in awareness of and willingness to participate in cancer clinical trials between African American and White cancer survivors

**DOI:** 10.1186/s12885-022-10082-9

**Published:** 2022-09-15

**Authors:** Gaurav Kumar, Jungyoon Kim, Paraskevi A. Farazi, Hongmei Wang, Dejun Su

**Affiliations:** 1 Center for Reducing Health Disparities, Department of Health Promotion, College of Public Health, University of Nebraska Medical Center, 984340 Nebraska Medical Center, Omaha, NE 68198-4340 USA; 2 Department of Health Services Research and Administration, College of Public Health, University of Nebraska Medical Center, Omaha, NE USA; 3 Department of Epidemiology, College of Public Health, University of Nebraska Medical Center, Omaha, NE USA

**Keywords:** Cancer clinical trial, Cancer survivors, Awareness, Willingness, Perception, Racial disparity

## Abstract

**Background:**

Cancer clinical trials (CCTs) are essential for cancer care, yet the evidence is scarce when it comes to racial disparities in CCT participation among cancer survivors in the Midwest. This study aimed to 1) assess disparities in the awareness of and willingness to participate in CCTs between African American and White cancer survivors; and 2) compare perceptions about CCTs between the two racial groups.

**Methods:**

The study was based on cross-sectional data from the survey “Minority Patient Participation in Cancer Clinical Trials” that collected information from 147 Black and White cancer survivors from Nebraska between 2015 and 2016. Chi-square tests and logistic regressions were used to assess differences between Black and White cancer survivors regarding their awareness, willingness, and perceptions associated with CCT participation.

**Results:**

After adjusting for the effects of socio-demographic, health status, and psychosocial variables, Black cancer survivors were much less likely than White cancer survivors to be aware of CCTs (AOR 0.26; CI 0.08–0.81), to express willingness to participate in CCTs (AOR 0.03; CI 0.01, 0.32) and to actually participate in CCTs (AOR 0.13; CI 0.04–0.38). Black cancer survivors reported a lower level of trust in physicians and were less likely than White cancer survivors to believe that CCTs make a significant contribution to science.

**Conclusions:**

Relative to White cancer survivors, Black cancer survivors had much lower awareness of and willingness to participate in CCTs. Part of these differences might be related to the differential perception of CCTs, psychosocial factors, and trust in physicians between the two groups.

## Introduction

Cancer clinical trials (CCTs) are crucial for developing and testing new treatments for cancer patients. However, only 3 to 5% of eligible adult cancer patients participate in clinical trials, even though most Americans (70%) view clinical trial participation favorably [1, 2]. Low enrollment in CCT delay cancer research advancement and increases the costs of developing and disseminating effective cancer treatments [3, 4]. In the United States (US), racial and ethnic minorities bear a disproportionate burden of cancer morbidity and mortality relative to non-Hispanic Whites [5, 6]. Less than 2% of the National Cancer Institute (NCI) sponsored clinical trials focused on racial/ethnic minorities, a proportion significantly lower than their percentage of the US population (36.3%) [6]. Despite the disparities, racial and ethnic minorities, particularly African Americans [3, 7–9], are consistently underrepresented in CCTs. The enrollment fraction (the number of trial enrollees divided by the estimated US cancer cases) in clinical trials by race/ethnicity for all cancers was 1.8% for Whites and 1.3% for African Americans [6].

Racial differences in CCT participation can be attributed to various factors, including but not limited to socioeconomic status (SES), logistical barriers, perception of, and attitude towards clinical research [1, 10]. Studies have identified several reasons for lower clinical trial participation among minority populations, including distrust of health care institutions, fear of experimentation, lack of awareness of trials and how to find them, limited representation of minority investigators, and poor communication with physicians [7, 11]. Based on a systematic review of the related literature, one of the most cited conceptual models (Ford Model) focused on three critical barriers to recruiting underrepresented cancer patients to CCTs, including awareness, opportunity, and acceptance [8, 12]. This conceptual model identified barriers to trial awareness including lack of education, limited culturally appropriate information, inadequate cancer knowledge, and physician awareness of trials [8, 9, 12]. Significant barriers for the opportunity to participate were old age, low SES, racial and ethnic minority status, study eligibility and exclusion criteria, provider attitudes, limited provider referral, and patient-provider communication regarding trials [8, 12]. Common barriers to acceptance of trial enrollment based on the Ford Model included mistrust of the research, perceived harms of clinical trial participation, time commitment, income, and transportation [12–14]. Moreover, fatalism, low self-efficacy, and limited social support are also associated with lower participation in CCTs [1, 2, 11].

Racial disparities in CCT participation reflect significant structural barriers many African Americans have to overcome before their participation rate can be increased. Poverty and living in under-resourced areas have made it difficult for African Americans to access care provided by teaching hospitals where CCTs are usually offered. Meanwhile, the underrepresentation of African American professionals in oncological care [15] also poses a challenge for delivering culturally sensitive care to African Americans and alleviating their mistrust in medical research in light of the painful memories of inappropriately conducted clinical trials such as the Tuskegee Syphilis Study [16]. Perceived or real experience of racism was identified as an important source of mistrust in healthcare professionals by African Americans, which is closely related to their lack of willingness to participate in medical research [17].

Previous research has revealed that compared with White Americans, African Americans were not only less likely to be aware of clinical trials [7, 18], they were also less willing to participate in clinical trials [19]. Despite the importance of these findings, one limitation is that most extant studies were based on data collected from the general population, not from cancer survivors. Related evidence is more scarce among cancer survivors in the Midwest. The present study’s primary objective is to examine differences in awareness of and willingness to participate in CCTs among Black and White cancer survivors from Nebraska. A secondary objective is to compare differences in perceptions of CCTs between Black and White cancer survivors.

## Methods

### Study setting and study population

This study used the data collected as part of a cross-sectional survey entitled “Minority Patient Participation in Cancer Clinical Trials,” conducted by the Center for Reducing Health Disparities at the University of Nebraska Medical Center in Omaha, Nebraska, from 2015 to 2016. The research team recruited a purposive sample of 176 cancer survivors who lived in Omaha, the largest city in Nebraska. We limited the sample of this study to 147 participants who self-identified as being White or African American for our analyses. Other races and multiracial and multiethnic individuals were excluded (*n* = 29). Our working sample consisted of approximately 84% of the full data set.

The research team distributed anonymous paper-based surveys at several local hospitals, clinics, community health centers, and a community event supporting African American female breast cancer survivors. Participants were eligible if they were 19 years or older and were current cancer patients or survivors. Participation in the survey was voluntary based on informed consent shared with potential participants at the beginning of the survey. The survey was completed by the participant themselves or with the help of research assistants when needed. All participants received a $20 gift card for compensating their time for their participation in the survey. Approvals for the study were obtained from the University of Nebraska Medical Center Institutional Review Board (IRB# 722–15-EX) before the initiation of data collection.

### Measurements

The research team developed and pilot-tested the survey questionnaire in the team before administering the survey. The questionnaire included basic demographic information, current cancer treatment status, awareness, and perception of CCT, experience in CCT participation, access to health services, and psychosocial constructs on social support, general self-efficacy, fatalism, and hopefulness.

#### Outcome variables

The primary outcome variables for our analyses are awareness of and willingness to participate in a CCT. Awareness of a CCT was assessed by a yes-no response to the question, “Have you heard about cancer clinical trials?” The willingness to participate in cancer clinical trials was measured by two questions. The first question “Would you ever participate in a cancer clinical trial?” was asked among all respondents who were provided with three options, including yes and no/unsure. The other question was only asked among those respondents who indicated that they had been offered to participate in a CCT, “Did you accept the offer to participate in the cancer clinical trial?” with three response options including yes, no/unsure.

Participant perception of CCT was based on response to the following questions: 1) “If your physician recommends that you participate in a cancer clinical trial, do you trust that he or she would fully explain it to you?” 2) “Do you believe you can freely ask your physician any questions about cancer clinical trials?” 3) “How often, if ever, do you think physicians prescribe medication as a way of experimenting on people without their knowledge or consent?” 4) “What are some of the features you would expect from a good cancer clinical trial program?” 5) “I have a good understanding about how clinical trials work” (strongly agree, agree, neutral, disagree, strongly disagree). 6) “Do you feel like clinical trials make a significant contribution to science (strongly agree, agree, neutral, disagree, strongly disagree)?” While the first three questions focused on patient trust in physicians, the fourth question assessed patient preferences for CCTs, and the final two questions evaluated understanding and knowledge of CCTs.

#### Explanatory variables

##### Demographics, socioeconomic status, and health

Basic demographic information included sex (male, female) and self-reported race (Whites and African American). Socioeconomic status was characterized by education level (up to high school, some college or more), marital status (married; non-married), employment status (employed, non-employed), and individual annual income (≤ $49,999, ≥ $50,000). Indicators on health included self-rated health status (high, low), physical activity status (active, non- active), and current cancer treatment status (yes, no).

##### Multidimensional Scale of Perceived Social Support (MSPSS)

This is a 12-item measure of social support’s perceived adequacy from three sources: family, friends, and significant other using a 5-point Likert scale (0 = strongly disagree, 5 = strongly agree). Mean score ranging from 1 to 2.9 suggests low support; a score of 3 to 5 indicates moderate support, and a score from 5.1 to 7 could be considered high support [20]. The Cronbach’s alpha of MSPSS was 0.96 in the current sample, suggesting very good internal consistency.

##### Fatalism scale

This scale measures cancer fatalism and comprises of three dimensions: predetermination, luck, and pessimism. The scale consists of 20 items with an overall score ranging from 20 to100 based on five-point Likert scales, with a higher score indicating a higher level of fatalism. The scale is reliable and valid for measuring fatalism among cancer patients [21]. Internal consistency reliability was estimated with Cronbach’s alpha (α) of 0.90 in the current sample.

##### General Self-Efficacy scale (GSE)

This 10-item self–assessment scale about the general belief in oneself to solve problems and reach goals. The scale ranges from 10 to 40, with higher scores denoting better self-efficacy or confidence in your ability to manage an illness or follow through with behavior change successfully. It has well-established validity and reliability [22], and in the present study, Cronbach’s alpha is 0.89.

##### Herth Hope index

This is a 12 item self-assessment scale measures various dimensions of hope using a 4-point Likert scale that ranges from 1 (strongly disagree) to 4 (strongly agree). The scale has one global score that ranges from 12 to 48, with higher scores associated with more hope. The scale is valid and reliable [23], and in the current study, the estimated Cronbach’s alpha was 0.85.

### Statistical analysis

We first used descriptive statistics to profile the sample and to compare demographics and health characteristics between Black and White cancer survivors. Means and standard deviations were calculated for continuous variables, whereas categorical variables were summarized with frequencies and percentages. Chi-square tests or Fisher’s exact tests were used to assess differences between groups for categorical variables, and t-tests were used to evaluate differences with respect to continuous variables. For multiple response variables, we used Cochran’s Q test to assess group differences [24]. Multivariable logistic regressions were estimated to examine racial differences in the odds of being aware of a CCT or willing to participate in it after adjusting for relevant confounders. Two-sided *p* values of less than 0.05 were considered statistically significant. The statistical analysis was performed using the IBM Statistical Package for Social Sciences for Windows, Version 25.0.

## Results

Racial differences in selected covariates included in our analysis are shown in Table 1. Out of the 147 cancer survivors, 95 (or 65%) were Black, and 52 (or 35%) were White. There was an obvious gender imbalance in the sample with 86% being female. Black and White cancer survivors differed significantly with respect to socioeconomic characteristics. Relative to White cancer survivors, African American cancer survivors were less likely to be married (41% vs. 71%; *p* <  0.001) and had a lower level of education, with 59% having completed some college or more as compared to 83% among White cancer survivors (*p* <  0.001). White cancer survivors had higher incomes and a higher proportion currently undergoing cancer treatment. Another notable difference was that Black cancer survivors showed a higher level of fatalism compared to White cancer survivors (*p* = 0.011). The two racial groups had similar employment status, self-rated health status, and physical activity levels.

**Table 1:** Sample description by race (number (%) or mean (standard deviation))

Variables	Total (*n* = 147)	White (*n* = 52)	Black (*n* = 95)	*P*-value
Gender				0.011
Male	20 (14)	12(24)	8 (8)	
Female	126 (86)	39 (76)	87 (92)	
Educational Level				0.003
Up to high school	48 (33)	9 (17)	39 (41)	
Some college or more	99 (67)	43 (83)	56 (59)	
Marital Status				0.001
Married	76 (52)	37 (71)	39 (41)	
Non-married	71 (48)	15 (29)	56 (59)	
Employment Status				0.950
Employed	73 (51)	26 (50)	47 (51)	
Unemployed	72 (49)	26 (50)	46 (49)	
Individual income ($)				0.007
≤ 49,999	101 (71)	30 (58)	71 (79)	
≥ 50,000	41 (29)	22 (42)	19 (21)	
Self-Rated Health Status				0.686
High	102 (69)	35 (67)	67 (70)	
Low	45 (31)	17 (33)	28 (30)	
Physical Activity Status				0.831
Active	114 (81)	42 (84)	72 (81)	
Non-Active	26 (19)	9 (18)	17 (19)	
Current treatment status				0.008
Yes	39 (27)	21 (40)	18 (20)	
No	104 (73)	31 (60)	73 (80)	
Years survived after initial cancer diagnosis, mean (S.D.)	10.85 (9.89)	7.05 (7.73)	13.25 (10.39)	0.007
Social Support Scale, mean (SD)	4.16 (0.84)	4.25 (0.67)	4.11 (0.91)	0.318
Fatalism Scale, mean (SD)	2.32 (0.61)	2.18 (0.55)	2.47 (0.67)	0.011
General Self Efficacy Scale, mean (S.D.)	3.21 (0.50)	3.24 (0.43)	3.19 (0.58)	0.579
Herth Hope Index, mean (SD)	3.12 (0.49)	3.09 (0.44)	3.16 (0.55)	0.467

Not all columns add up to *n* = 147 due to missing value. Chi-square tests or Fisher’s exact tests were conducted for categorical variables, and t-tests were used for continuous variables

### Racial differences in awareness of and willingness to participate in CCTs

When asked whether they had ever heard about cancer clinical trials, 63% of the respondents answered positively (Table 2). Cancer clinical trials’ awareness was much higher among white cancer survivors than among African Americans (80% vs. 52%), and the difference was statistically significant (*p* = 0.001).

**Table 2:** Awareness of and Willingness to Participate in CCT by Race

Variables	Total n (%)	White n (%)	Black n (%)	*P*- value
Have you heard about CCT?				0.001
Yes	85 (62.5)	40 (80.0)	45 (52.3)	
No	51 (37.5)	10 (20.0)	41 (47.7)	
Would you ever participate in CCT?				< 0.001
Yes	59 (42.1)	35 (68.6)	24 (27.0)	
No/Unsure	81 (57.9)	16 (31.4)	65 (73.0)	
Did you accept the offer to participate in CCT?				< 0.001
Yes	25 (30.5)	15 (57.7)	10 (16.9)	
No/Unsure	60 (69.5)	11 (42.3)	49 (83.1)	

*P* values were based on Chi-square tests of the bivariate associations between race and selected variables

When asked whether they would be willing to participate in a CCT, overall, 42% of the respondents were willing, and 58% were unsure or were not interested in participation. However, there were significant differences in willingness to participate between Black and White cancer survivors. Sixty-nine percent of White cancer survivors were willing to participate in a CCT, as compared to 31% of African Americans (*p* <  0.001). Among all respondents who reported that they had been offered the opportunity to participate in a CCT, 31% reported that they accepted the offer to participate. This percentage of ever participated in a CCT was much higher among White cancer survivors (58%) than among Black cancer survivors (17%) (*p* <  0.001).

Table 3 displays the adjusted odds ratios (AOR) of significant factors associated with awareness of and willingness to participate in cancer clinical trials. After adjusting for the effect of selected variables on demographics, socioeconomic status, self-rated health, physical activity status, current cancer treatment status, and psychosocial characteristics, Black cancer survivors were much less likely than White cancer survivors to be aware of a CCT (AOR 0.26; CI 0.08–0.81), to express willingness to participate in a CCT (AOR 0.13; CI 0.04–0.38), and to actually accept the offer to participate in a CCT (AOR 0.03; CI 0.01–0.32).

**Table 3:** Logistic regressions on awareness of and willingness to participate in CCT among White and Black cancer survivors (adjusted odds ratio & 95% confidence interval)

Race	Awareness of CCT	Expressed Willingness to Participate in CCT	Actual Participation in CCT
White	1.00	1.00	1.00
Black	0.26^*^ (0.08, 0.81)	0.13^**^ (0.04, 0.38)	0.03^**^ (0.01, 0.32)

Adjusted odds ratios associated with race were estimated after controlling for the effect of gender, educational level, marital status, employment status, individual income, self-rated health status, physical activity status, current status of treatment, years survived after initial cancer diagnosis, social support, fatalism, general self-efficacy and hope index

^*^p < 0.05

^**^p < 0.01

### Racial differences in perception of cancer clinical trials

When asked, “If your physician recommends that you participate in a cancer clinical trial, do you trust that he or she would fully explain it to you?”, 96% of White cancer survivors and 86% of African American cancer survivors in the study responded yes (*p* = 0.06) (Table 4). In both groups, over 90% thought they could freely ask physicians any questions about the cancer clinical trials (*p* = 0.71). However, an important difference between the two groups lies in their responses to the question “How often, if ever, do you think physicians prescribe medication as a way of experimenting on people without their knowledge or consent?” While 34% of African American cancer survivors believed this was done very or fairly often, only 6% of the White cancer survivors believed this was the case (*p* < 0.001).

**Table 4:** Perception of CCT by race

Survey Questions	Total n (%)	White n (%)	Black n (%)	***P***-value
If your physician recommends that you participate in a cancer clinical trial, do you trust that he or she would fully explain it to you?				0.06
Yes	122 (90)	48 (96)	74 (86)	
No	14 (10)	2 (4)	12 (14)	
Do you believe you can freely ask your physician any questions about cancer clinical trials?^a^				0.710
Yes	127 (94)	48 (96)	79 (93)	
No	8 (6)	2 (4)	6 (7)	
How often, if ever, do you think physicians prescribe medication as a way of experimenting on people without their knowledge or consent?				0.001
Very/ Fairly often	33 (24)	3 (6)	30 (34)	
Rarely/Never	67 (49)	34 (69)	33 (38)	
Do not know	26 (27)	12 (25)	24 (28)	
What are some of the features you would expect from a good cancer clinical trial program (select all that apply)?^a^				0.001
Send me the educational information through mails	99 (74)	39 (29)	60 (45)	
Online information about the program	73 (55)	41 (31)	32 (24)	
Offer flexible scheduling	84 (63)	39 (29)	45 (34)	
Close to where I live	85 (63)	32 (24)	53 (40)	
A physician with a similar cultural background as me	32 (24)	6 (5)	26 (19)	
Communication technology including telemedicine	65 (49)	28 (21)	37 (28)	
Others	12 (9)	6 (5)	6 (5)	
I have a good understanding about how clinical trials work.				0.090
Strongly disagree/Disagree	44 (33)	11 (22)	33 (39)	
Neutral	42 (31)	16 (32)	26 (30)	
Strongly Agree/Agree	49 (36)	23 (46)	26 (30)	
Do you feel like clinical trials make a significant contribution to science?				0.015
Strongly disagree/Disagree	13 (10)	2 (4)	11 (13)	
Neutral	25 (19)	5 (10)	20 (24)	
Strongly Agree/Agree	95 (71)	43 (86)	52 (63)	

^a^Multiple response variable and Cochran’s Q *p* value used

The two groups of cancer survivors also showed differences in their preferences of desired features of a good cancer clinical trial program. The most preferred feature for African Americans was that the program could send the trial’s educational information through mails, followed by having the trial close to residence. By contrast, the most preferred feature for White cancer survivors was providing online information about the program, followed by flexible scheduling and sending educational information about the trial through mails.

In terms of understanding and knowledge of CCTs, 46% of Whites and 30% of African Americans in the sample believed that they had a good understanding of how clinical trials worked (*p* = 0.09). However, a more pronounced difference between the two groups was their opinion on whether clinical trials make a significant contribution to science. Eighty-six percent of White cancer survivors believed that clinical trials make a substantial contribution to science, as compared to 63% among African American cancer survivors (*p* = 0.015).

## Discussion

The underrepresentation of African American patients in CCTs remains a challenge for developing personalized, precision medicine in cancer therapy. According to the U.S. Federal Drug Administration, out of the 5157 patients who participated in clinical trials that led to the approvals of 17 new cancer drugs in 2018, only 4% of them were African American patients [25]. Based on survey data collected from cancer survivors in Nebraska, our findings in the current study point to several significant barriers that have hindered CCT participation among African American cancer patients, including lack of awareness of CCTs, lack of willingness to participate in CCTs, distrust in physicians, and prevailing misconceptions of CCTs.

Being aware of clinical trials is necessary for cancer patients to make informed decisions on trial participation. The substantial gap in CCT awareness between White and African American cancer survivors, as revealed in this study (80% vs. 52%), underscores the need to identify unique barriers encountered by African American cancer patients in knowing about CCTs. While part of these barriers might be related to the relatively lower level of education and health literacy among African Americans, our study’s findings suggested that adjusting for the effect of education and other socioeconomic status variables did not substantially alter the originally observed racial gap in CCT awareness. Multiple studies have shown that African Americans have a general lack of grasp of the research process and medical terms like clinical trials and medical research [26, 27]. A study that looked at cancer health literacy among African Americans, Whites, and Hispanics backed with similar findings. On a cancer health literacy test, African American participants scored much lower than white participants [28]. The relatively lower level of health literacy among African Americans could lead to problems in understanding the informed consent process in biomedical research, a common obstacle to African American involvement in CCT [27, 29]. Another qualitative study interviewing African American cancer survivors at a safety-net hospital identified that a lack of understanding of cancer clinical trials was one of the major barriers of participation in cancer trials [28, 30]. The study reported that many survivors were confused and even could not be differentiated between a cancer clinical trial and treatment of cancer [31]. Our findings are consistent with these studies and suggest the need to provide culturally and linguistically appropriate cancer clinical trial information to AA cancer survivors to ensure understanding of important clinical trial terms and concepts. Some of the evidence-based strategies include a culturally tailored educational video or in-person education [32, 33], patient-focused, relationship-building communication strategies [34], or the use of patient navigators [35].

Since physicians are the primary source of information for patients to know about CCTs [36–38], it would be also important to examine CCT awareness among care providers serving African American patients and whether these care providers would inform African American patients about CCTs. There was evidence that oncologists’ visits with African American patients, on average, were shorter compared with visits with White patients and included less discussion of the purpose and risks of trials offered but more discussion of voluntary participation. As a result, African American patients may make decisions about clinical trial participation based on less discussion with oncologists than do White patients [37–39].

Besides limited awareness of cancer trials, Black cancer survivors also demonstrated much less willingness to participate in the trials. Among the 57 Black cancer survivors in this study who had been offered to participate in a CCT, only 10 of them, or 18%, accepted the offer compared to 60% among White cancer survivors. Closely related to the lower participation rate in Black cancer survivors were the observation that, relative to White cancer survivors, Black cancer survivors reported a higher level of distrust in physicians and misconceptions of CCTs. Previous studies also documented that Black cancer patients were more likely than their White counterparts to express distrust in CCTs [39, 40]. Besides the trust issue, the knowledge gap in understanding CCTs between the two racial groups should also be addressed to encourage CCT participation among African American cancer survivors. Cancer patients are usually more likely to participate in CCTs when they are convinced of the treatment efficacy or how the trial results could advance cancer treatments and benefit other patients. According to findings from one recent study [40], the top two reasons patients cited for their participation in CCTs were 1) ‘the trial offered the best treatment available’ and 2) that ‘the trial results could benefit others.’ This study concluded that patients’ motivations for trial participation included perceived personal benefit and altruistic reasons. Effective and culturally appropriate communication between care providers and African American cancer patients is important and needed to reduce distrust and improve patient understanding of clinical trials [41, 42]. Studies on perceptions of clinical trials among racial and ethnic minorities have consistently shown the importance of developing culturally specific assessments for these perceptions and tailoring educational strategies to correct misconceptions [19, 43–46].

Moreover, our study findings showed a higher level of fatalism and hopefulness, lower self-efficacy, and social support among African Americans when compared to White Cancer survivors. This difference could be explained by the substantial gap in African American participants’ socio-demographic characteristics such as gender, educational level, marital status, and income compared with Whites. These factors could also influence the racial differences in awareness of and willingness to participate in the CCT in the given sample. Studies have suggested that cancer fear, cancer fatalism, along with limited social support, and self-efficacy have a direct or indirect influence on the cancer care continuum, awareness of and willingness to participate in CCTs [2, 39, 42].

This study provides some clues on designing future CCTs that might appeal to African American cancer patients. The top three desired features of CCTs identified by African American cancer patients in the study included sending information about the trial through mails, having the trial close to residence, and offering flexible scheduling for participation, which were somewhat different from the features preferred by White cancer survivors in the study. Meanwhile, African American cancer survivors were more likely than White cancer survivors to prefer having a physician with a similar cultural background (19% vs. 5%). There was also evidence that relative to Whites, African Americans were more likely to look to their churches for clinical trial information, whereas Whites were more likely to seek information from a doctor or the Internet [19]. These differences between the two racial groups reinforce the need to develop tailored strategies for recruiting diverse groups of cancer patients into clinical trials. Enrolling individuals with specific cultural backgrounds and literacy levels may help to increase the recruitment of underrepresented groups (African Americans). Patient navigation models, which involve delivering educational and facilitative services to patients, have been proposed to promote the retention of African Americans in cancer clinical trials, with one study indicating a 9 to 16% increase in participation [45]. Attempts to lessen the influence of bias in clinical interactions could be another answer. Training healthcare providers in the use of high-quality patient-centered communication are one possibility. Health care providers must be culturally aware and exhibit proper communication skills in order to successfully enroll African Americans in clinical trials [44, 45]. Additionally, leveraging a community’s resources and assets while conducting collaborative research and building equitable partnerships has the potential to strengthen community buy-in and engagement in CCT [46].

### Study limitations and strengths

Several limitations of our study are noteworthy in light of the results. First, the cross-sectional design, in conjunction with our reliance on self-report of clinical trial participation, can lead to potential recall bias. Secondly, our use of a convenience sample in Nebraska with oversampling of African American cancer survivors calls for caution when generalizing findings from the study to other cancer survivor populations. In particular, our success in recruiting many African American breast cancer survivors from a major annual event hosted by My Sister’s Keeper, our community partner in this study, has resulted in the overrepresentation of females and African American breast cancer survivors in this study. Future studies can assess the robustness of our findings in more representative samples of cancer survivors. Thirdly, some measures used in this study such as awareness of CCTs, willingness to participate in CCTs, and trust in physicians, were developed by the study team, not based on extant, validated measures from the literature. Their validity and reliability need to be further assessed before being adopted in other studies. Finally, due to data constraints, patient perception of CCTs was based on the analysis of multiple-choice questions in the survey, not qualitative feedback from patients, which has limited the depth of the analysis. Despite these limitations, to our knowledge, the study represents a rare effort that has revealed substantial gaps in awareness of and willingness to participate in CCTs between White and Black cancer survivors in the Midwest with a large rural population, and related racial differences in the perceptions of clinical trials and trust in physicians. The identified differences in the desired features of clinical trials between Black and White cancer survivors in this study might help facilitate the development of future clinical trials that can more effectively engage cancer patients.

## Conclusions

Relative to White cancer survivors, African American cancer survivors were much less likely to be aware of or to participate in CCTs. These disparities persisted with or without adjusting for racial differences in demographics, SES, health status, psychosocial status (hopefulness, fatalism, self-efficacy), and other factors considered in this study. Consistent with these disparities, African American cancer survivors reported a lower level of trust in physicians and a more deficient understanding of CCTs. Developing tailored, evidence-based strategies to more effectively engage African American cancer survivors and increase their awareness of and willingness to participate in CCTs would be crucial for improving the representativeness of minority patients in CCTs and for addressing racial disparities in CCT participation.

## Data Availability

Study data have been archived at the Center for Reducing Health Disparities, University of Nebraska Center, and can be requested by contacting Dr. Dejun Su via email at dejun.su@unmc.edu.

## Abbreviations

AOR: Adjusted odds ratio
CCT: Cancer clinical trial
CI: Confidence interval
SES: Social economic status
